# Effect of Myo‐Inositol Treatment on Serum Asprosin Levels of Pregnant Women: A Prospective Randomized Controlled Pilot Study

**DOI:** 10.1155/jp/8816154

**Published:** 2025-12-28

**Authors:** Ali Cenk Özay, Özlen Emekçi Özay, Oğuz Han Edebal, Yusuf Özay, Mario Montanino Oliva, Simona Dinicola, Vittorio Unfer

**Affiliations:** ^1^ Department of Obstetrics and Gynecology, Cyprus International University, Nicosia, Cyprus, ciu.edu.tr; ^2^ The Experts Group on Inositol in Basic and Clinical Research and on PCOS (EGOI-PCOS), Rome, Italy; ^3^ Department of Biochemistry, Near East University, Nicosia, Cyprus, neu.edu.tr; ^4^ Department of Medical Biology, Adıyaman University, Adıyaman, Turkey, adiyaman.edu.tr; ^5^ Department of Obstetrics and Gynecology, Santo Spirito Hospital, Rome, Italy; ^6^ Systems Biology Group Lab, Rome, Italy; ^7^ Department of Gynecology and Obstetrics, UniCamillus-Saint Camillus International University of Health Sciences, Rome, Italy

**Keywords:** asprosin, gestational diabetes, insulin resistance, myo-inositol, pregnancy

## Abstract

**Objectives:**

Asprosin is a newly discovered adipokine associated with insulin resistance and diabetes mellitus. Currently, its role during gestation is under investigation, as asprosin seems to increase during pregnancy, contributing to the onset of complications, like gestational diabetes. Considering the beneficial effects of myo‐inositol to support the physiological pregnancy, recovering and preventing adverse maternal and fetal outcomes, we aimed to evaluate the effects of its supplementation on serum asprosin levels in pregnant women.

**Design:**

We enrolled 40 patients at the early stages of pregnancy and randomly distributed them to a study group, which received 2‐g myo‐inositol and 200‐*μ*g folic acid twice a day, or to a control group, which received the sole folic acid.

**Results:**

After 20–22 weeks of treatment, we recorded a decrease of serum asprosin values as well as of HOMA‐IR index in the group supplemented with myo‐inositol, while the group that took only folic acid showed an increase in asprosin levels and no worsening of insulin resistance indices (HOMA‐IR index).

**Limitations:**

The small number of patients could be a limitation of the study.

**Conclusions:**

Asprosin may be modulated by myo‐inositol. This opens the possibility of considering this adipokine as a useful marker of insulin resistance to assess in pregnant women and to efficaciously target in clinical practice.

**Trial Registration:** ClinicalTrials.gov identifier: NCT05943158.

## 1. Introduction

Asprosin is a pluripotent adipokine mainly synthesized and released by the white adipose tissue (WAT) during fasting. In particular, plasma levels of asprosin increase after overnight fasting and decrease after feeding. It derives from the gene of profibrillin and its first mention occurred in 2016 by Romere et al. [1]. Such a molecule exerts a complex role in the central nervous system, appetite regulation, glucose metabolism, and insulin resistance [2]. Besides demonstrating that asprosin stimulates glucose secretion from the liver, subsequent preclinical and clinical studies described elevated serum levels of asprosin in pathological conditions, such as insulin resistance with polycystic ovary syndrome, Type 2 diabetes mellitus (T2DM), obesity, and metabolic syndrome [1, 3–5]. Pregnancy is a diabetogenic process during which insulin resistance may also increase [6]. Indeed, one of the most important metabolic changes in pregnancy regards the production of insulin which helps to promote maternal nutrient storage to support the energy demands of the fetus. Also, the production of some adipokines increases during pregnancy [7]. In particular, other adipokines, such as leptin, visfatin, and adiponectin, are dysregulated in gestational diabetes (GDM) and they may have a pathological significance and prognostic value in this pregnancy disorder. Considering the recent discovery of asprosin, a still limited number of studies investigated the correlation between pregnancy and asprosin, which is produced also from the placenta. Studies examining asprosin levels in pregnancy mostly evaluate the relationship with the occurrence of GDM. This latter—defined as glucose intolerance with variable severity of hyperglycemia—is the most common metabolic disorder during pregnancy and its prevalence varies between 5% and 10% according to the examined patient population and the used diagnostic test [8–10]. In addition, these patients also have impaired insulin secretion [6], and recent studies reported increased levels of asprosin in the blood of patients with GDM [11, 12]. Indeed, in the pathogenesis of GDM, the adipose tissue seems to play a crucial role. It is considered to be an endocrine organ that, through the production of adipokines, regulates many biological functions and influences pregnancy as well as several pregnancy complications, such as GDM [13]. Inositol is a polyol structure molecule belonging to the vitamin B complex; it is produced in different organs in the body or supplemented from the outside with food [14, 15]. Among the nine inositol stereoisomers, myo‐inositol (MI) is one of the most important and studied in recent years. Pieces of evidence demonstrated that starting inositol supplementation in the early weeks of pregnancy prevents GDM onset, especially in those women with a high risk of developing GDM like those with obesity and polycystic ovary syndrome [16, 17]. MI, which is an intracellular second messenger with insulin‐like effects on glucose metabolism, also improves insulin resistance [18]. Based on data in the literature, altered serum asprosin levels during pregnancy may play a crucial role in the pathogenesis of GDM. Therefore, considering the positive effects of MI on glucose metabolism and GDM and, on the other hand, the increased levels of asprosin in conditions of glucose alterations, in this study, we aimed to evaluate the effect of MI on serum levels of asprosin in pregnant women starting the oral administration of MI in the early stages of pregnancy. The overall goal is to verify whether the protective effect of MI against GDM is associated with reduced plasma levels of asprosin, thus correlating for the first time these two molecules.

## 2. Materials and Methods

### 2.1. Study Design, Patients, and Treatment

**Figure 1:**
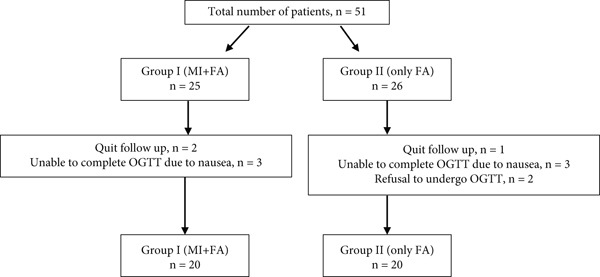
Flowchart of the study.

This prospective study was carried out on women who applied to the Near East University Medical Faculty Hospital Gynecology and Obstetrics outpatient clinic and agreed to participate in the trial between 1 June 2021 and 30 June 2022. All the enrolled patients gave their informed consent after the explanation of the study purpose. The study was conducted following the Ethical Principles of the Declaration of Helsinki, and ethics committee approval was obtained from the local ethics committee (Ethical Approval Number YDU/2021/90‐1334, first registration 13/07/2023). The study population consisted of 40 pregnant patients between the ages of 18 and 40. Sample size calculation was performed using G∗Power 3.1. For *α* error probability of 0.05 and 0.8 power with an effect size of 0.95, the sample size should be at least 20 in each group. Demographic characteristics (age, height, weight, gravida, and parity) were recorded, and all participants underwent an ultrasonographic evaluation at 5–6 weeks of gestation (baseline) and at the gestational week of the oral glucose tolerance test (OGTT). During the first trimester, all the pregnancy routine blood tests were performed, fasting insulin and fasting glucose (FG) values were recorded, and insulin resistance was calculated for all the patients. Besides the pregnancy routine baseline blood analysis between 5 and 6 weeks of gestation, an extra tube of blood was taken for the analysis of serum levels of asprosin: sera were separated and stored at −80° until the day of analysis. Afterwards, patients were randomized according to the application protocol number: Group 1 took 2 g MI + 200 *μ*g folic acid twice a day (Inofolic, Lo.Li. Pharma s.r.l., Rome, Italy) while Group 2 took 200 *μ*g folic acid twice a day. The treatment time was between 20 and 22 weeks for both groups. Allocation was randomly defined with an Excel′s basic random number generator. The same clinical guidelines were followed for all pregnant women enrolled in the study in terms of diet, physical activity prescription, number of medical check‐ups, etc. Recruitment was stopped when the number of participants in each group reached 20. The flowchart of the study is given in Figure 1. Exclusion criteria were the occurrence of chronic hypertension, prepregnancy diabetes or a history of GDM in a previous pregnancy, assumption of insulin sensitizers such as metformin, multiple pregnancy, history of pregnancy‐induced hypertension, addiction such as smoking or alcohol, and thyroid or other endocrine diseases.

### 2.2. Outcomes

Between the 24 and 28 weeks of gestation, an OGTT was performed by administering 75‐g glucose and another extra tube of blood was collected; the sera were kept at −80° until the analysis of asprosin levels. In this examination at 24–28 weeks of gestation, weight and fasting insulin values were recorded as in the first examination. For evaluating insulin resistance, the homeostasis model assessment‐insulin resistance (HOMA‐IR) was used. HOMA‐IR was calculated by multiplying fasting blood glucose and fasting insulin levels and dividing by 405, whereas the body mass index (BMI) was calculated based on the ratio of weight to height in square meters. HOMA‐IR and BMI were calculated both at the first examination, between 5 and 6 weeks of gestation (T0), and at the time of the OGTT between 24 and 28 weeks of gestation (T1). In addition, the onset of GDM, the gestational week, and the fetal weight at birth were recorded.

### 2.3. Statistical Analysis

The Statistical Package for the Social Sciences (SPSS) v.15 for Windows (SPSS Inc., Chicago, United States) was used for all the statistical analyses. The demographic data and test results were presented as mean ± standard deviation. The Mann–Whitney *U* test was used for the comparisons between groups, and the Wilcoxon test was used for the intragroup comparisons. A *p* value less than 0.05 was considered a statistically significant result.

## 3. Results

The obtained results were derived from 40 enrolled pregnant women. The groups were similar in terms of the gestational week at delivery, fetal birth weight, and demographic characteristics such as age, BMI, gravida, and parity **(**Table 1 **).**

**Table 1:** Comparison of demographic characteristics of Group 1 and Group 2.

	**Group 1 (** **n** = 20**)**	**Group 2 (** **n** = 20**)**	**p** **value**
Age	28.30 ± 4.30	28.70 ± 5.70	0.960
Pregnancy	1.50 ± 0.80	1.60 ± 0.70	0.562
Parity	0.30 ± 0.60	0.55 ± 0.60	0.204
Height (m)	1.63 ± 0.07	1.63 ± 0.06	1.000
Weight (kg)^a^	64.10 ± 12.90	62.50 ± 8.90	0.928
Weight (kg)^b^	75.00 ± 15.1	73.50 ± 9.80	0.849
BMI^a^	23.89 ± 3.61	23.38 ± 2.77	0.904
BMI^b^	27.98 ± 4.27	27.55 ± 3.33	0.992
Fetal birth weight (g)	3151 ± 312	3383 ± 451	0.089
Gestational week at birth	38.5 ± 1.1	38.30 ± 1.00	0.497

*Note:* Data is given as mean ± standard deviation. Mann–Whitney *U* test between Group 1 and Group 2.

Abbreviations: BMI, body mass index.

^a^Baseline measurements.

^b^Measurements at the gestational week of the oral glucose tolerance test.

The laboratory values of both groups at the first examination (T0) and at the gestational week of OGTT (T1) are shown in Table 2.

**Table 2:** Comparison of laboratory values between Group 1 and Group 2 at T0 and T1.

	**Group 1 (** **n** = 20**)**	**Group 2 (** **n** = 20**)**	**p** **value**
Asprosin (ng/mL)^a^	20.35 ± 15.42	24.88 ± 20.31	0.881
FG (mg/dL)^a^	77.60 ± 7.00	79.30 ± 8.90	0.624
Insulin (mU/L)^a^	7.30 ± 1.70	7.30 ± 2.00	0.992
HOMA‐IR^a^	1.39 ± 0.33	1.42 ± 0.35	0.681
Asprosin (ng/mL)^b^	17.90 ± 10.95	30.19 ± 25.71	0.317
FG‐OGTT (mg/dL)^b^	78.50 ± 9.00	82.50 ± 8.60	**0.041**
1‐h‐OGTT (mg/dL)^b^	126.50 ± 24.70	137.40 ± 44.50	0.674
2‐h‐OGTT (mg/dL)^b^	113.10 ± 21.80	116.50 ± 28.70	0.779
Insulin (mU/L)^b^	8.30 ± 4.70	9.30 ± 4.80	0.379
HOMA‐IR^b^	1.54 ± 0.57	1.89 ± 0.97	0.238

*Note:* Data is given as mean ± standard deviation. Mann–Whitney *U* test between Group 1 and Group 2. *p* < 0.05 is statistically significant (bold).

Abbreviations: FG, fasting glucose; HOMA‐IR, homeostasis model assessment‐insulin resistance; OGTT, oral glucose tolerance test.

^a^Baseline values.

^b^Values at the gestational week of the oral glucose tolerance test.

Only FG‐OGTT value was statistically significantly lower in the MI group, while other parameters were statistically similar.

Table 3 reports the changes in serum levels of asprosin, insulin, HOMA‐IR, weight, and BMI from the beginning of the supplementation until the time of OGTT between 24 and 28 weeks of gestation. In both groups, the rise in weight and BMI was statistically significant. Noteworthy serum asprosin values and HOMA‐IR index increased statistically only in Group 2 supplemented with folic acid. Even though without statistical significance, serum asprosin values decreased in Group 1 supplemented with MI + folic acid.

**Table 3:** Comparison of serum levels of asprosin, insulin, HOMA‐IR, weight, and BMI between Group 1 and Group 2.

	**Group 1 (** **n** = 20**)**			**Group 2 (** **n** = 20**)**		
	**Before**	**After**	**p** **value**	**Before**	**After**	**p** **value**
Asprosin (ng/mL)	20.35 ± 15.42	17.90 ± 10.95	0.603	24.88 ± 20.31	30.19 ± 25.71	**0.005**
Weight (kg)	64.10 ± 12.90	75.00 ± 15.10	**< 0.001**	62.50 ± 8.90	73.50 ± 9.80	**< 0.001**
BMI	23.89 ± 3.61	27.98 ± 4.27	**< 0.001**	23.38 ± 2.77	27.55 ± 3.33	**< 0.001**
Insulin (mU/L)	7.30 ± 1.70	8.30 ± 4.70	0.177	7.30 ± 2.00	9.30 ± 4.80	0.234
HOMA‐IR	1.39 ± 0.33	1.54 ± 0.57	0.219	1.42 ± 0.35	1.89 ± 0.97	**0.043**

*Note:* Data is given as mean ± standard deviation. Wilcoxon test at different time points within the same group. *p* < 0.05 is statistically significant (bold).

Abbreviations: BMI, body mass index; HOMA‐IR, homeostasis model assessment‐insulin resistance.

However, by evaluating the groups in terms of variation of the different parameters, serum levels of asprosin statistically decreased in the MI group compared to the folic acid group (*p* = 0.012) (Table 4).

**Table 4:** Variation (*Δ*) of serum asprosin, fasting glucose, insulin, HOMA‐IR, and weight in each group.

	**Group 1 (** **n** = 20**)**	**Group 2 (** **n** = 20**)**	**p** **value**
*Δ* asprosin	−2.451 ± 7.833	5.304 ± 11.380	**0.012**
*Δ* fasting glucose	0.9 ± 9.6	3.2 ± 10.4	0.698
*Δ* insulin	1.0 ± 4.1	2.0 ± 5.1	0.799
*Δ* HOMA‐IR	0.2 ± 0.5	0.5 ± 1.0	0.398
*Δ* weight	11.0 ± 5.6	11.0 ± 3.6	0.758

*Note:* Data is given as mean ± standard deviation. Mann–Whitney *U* test between *Δ* of Group 1 and *Δ* of Group 2. *p* < 0.05 is statistically significant (bold).

Abbreviation: HOMA‐IR, homeostasis model assessment‐insulin resistance.

While a significant rise in HOMA‐IR values was observed in Group 2 following treatment, the intergroup comparison of *Δ*HOMA‐IR revealed no significant difference. No patients experienced side effects related to supplementation. Pregnancy‐induced hypertensive disease did not occur among patients. Although the number of patients with a diagnosis of GDM was less in Group 1 after MI supplementation (3 patients in Group 1 and 5 patients in Group 2 were diagnosed with GDM), it was not statistically significant (*p* = 0.38).

## 4. Discussion

Asprosin is running as a new marker for insulin resistance. In this study, we demonstrated for the first time that the protective use of MI in pregnant women has a favorable effect also on asprosin levels, thus providing a possible way to modulate such adipokine. After the discovery of asprosin, several studies have investigated its contribution in different diseases and types of populations. Zhong et al. reported in their study that asprosin levels increased both in pregnant women with GDM and in related offspring more than in healthy mothers [19]. Yavuz et al. found higher asprosin levels in both milk and serum of pregnant women with GDM compared to the control group [20]. In their study, Baykus et al. found a statistically significant increase of asprosin levels in pregnant women with GDM and preeclampsia compared to the control group [12]. On the other hand, serum levels of asprosin in the cord blood were also close to maternal levels of asprosin [12]. Considering these data, serum asprosin levels are affected in pregnancy, which is itself a diabetogenic process. Indeed, changes in insulin sensitivity may occur during pregnancy and a growing number of studies suggested that other adipokines, including adiponectin, leptin, visfatin, resistin, and tumor necrosis factor *α*, are dysregulated in GDM and they might have pathological significance and prognostic value in this pregnancy disorder [13]. Evidence in the literature indicates the protective role of inositol derivatives in regulating glucose and insulin metabolism in both pregnant and nonpregnant women [16, 21]. Studies on healthy mice indicated that oral administration of MI may improve glucose tolerance correlating this effect with skeletal muscle translocation of glucose transporter 4 (GLUT4) to the plasma membrane, thus improving peripheral insulin sensitivity [22]. In a randomized placebo‐controlled study, women taking MI exhibited a significant improvement in HOMA‐IR values [23]. In the study of Corrado et al., pregnant women using MI for 8 weeks exhibited improved levels of glucose, fasting insulin, and HOMA‐IR [24]. Regarding the effect of MI in pregnant women, in their randomized controlled study, D′Anna et al. found a decreased insulin resistance in the group supplemented with MI for 2 months [25]. A 2015 Cochrane review stated that MI supplementation may be effective in reducing the incidence of GDM [26]. In a comprehensive review, MI supplementation, which is started in the early weeks of pregnancy, will also prevent the onset of GDM [27]. Recently, the results of a meta‐analysis including four studies declared that MI administration had positive effects on the incidence of GDM and improved levels of FG‐OGTT, 1‐h‐OGTT, and 2‐h‐OGTT [28]. Another trial provided evidence for the effectiveness of MI supplementation during pregnancy by reducing the incidence of GDM in pregnant women [29]. In our study, we examined the effect of MI on serum asprosin levels in healthy women without risk factors, starting the supplementation at the beginning of pregnancy. Serum asprosin at baseline and at the gestational week of OGTT did not show statistical significance between the two groups. However, when we analyzed the variation before and after the treatment, we found a significant improvement in the MI‐treated group. This data suggest that the oral administration of MI may have a favorable effect on asprosin levels, whose upregulation correlates with metabolic disorders such as GDM. Indeed, although without statistical significance—probably due to the small number of patients—GDM onset was less in the MI‐treated group compared to the control. In addition, considering that the number of patients with GDM was very small, we could not compare serum asprosin levels of participants according to the diagnosis of GDM. In our study, the FG‐OGTT value was significantly lower in the MI‐treated group compared to the control group in accordance with the literature; however, no significant difference occurred between the two groups in 1‐h‐OGTT and 2‐h‐OGTT values. The molecular mechanism of the improving effect of MI supplementation on serum asprosin levels is still unknown. Asprosin may stimulate hepatic glucose production by using cyclic adenosine monophosphate as a second messenger [1]. Asprosin and inositol may interact at the molecular level directly or indirectly. Considering the relationship between asprosin levels and insulin resistance [30], a possible link between inositol and asprosin could be via insulin pathways or they may interact via feedback mechanisms. In a recent study, it was indicated that MI promotes the transdifferentiation of adipose tissue from WAT to brown adipose tissue (BAT) [31]. Being asprosin produced by WAT, it is likely that the final effect (the reduction of asprosin levels) could be due to this conversion to BAT. Even though the small number of patients could be a limitation of the study, it is the first trial in the literature examining the effect of MI on asprosin levels in a randomized‐controlled study design with a homogeneous treatment and control group. Since the average BMI of the patients included in this study was within the normal range, the results of the study cannot be generalized to women with obesity. These data highlight for the first time the relationship between MI and asprosin levels opening the possibility to modulate asprosin levels as a therapeutic target in clinical practice.

## 5. Conclusions

By acting on insulin and glucose metabolism, MI supplementation reduces the incidence of GDM in pregnant women. In this study, we observed that starting MI supplementation in the early weeks of pregnancy has beneficial effects on serum asprosin levels, which is a new candidate as an insulin resistance marker. The molecular mechanism through which MI may influence asprosin levels is not yet known. Considering the recent discovery of asprosin, further studies are needed to deeply investigate and highlight the mechanisms underpinning its crucial role as a new insulin resistance marker.

## Ethics Statement

The study was conducted in accordance with the Declaration of Helsinki and approved by the Near East University Medical Faculty Hospital Gynecology and Obstetrics Ethics Committee YDU/2021/90‐1334.

## Consent

Written informed consent was obtained from all participants.

## Disclosure

All authors agree to be accountable for the content and conclusions of the article. No other persons or third‐party services, who are not listed as an author and have not been acknowledged, were involved in the research or manuscript preparation.

## Conflicts of Interest

S.D. and V.U. are employed at Lo.Li. Pharma Srl, Rome, Italy.

## Author Contributions

Conceptualization: Y.Ö. and A.C.Ö. Methodology: A.C.Ö. and Y.Ö. Software: A.C.Ö. and O.H.E. Validation: O.E.Ö., O.H.E., Y.Ö., and M.M.O. Formal analysis: Ö.E.Ö and A.C.Ö. Investigation: A.C.Ö. and Ö.E.Ö. Resources: A.C.Ö. and Ö.E.Ö. Data curation: A.C.Ö., S.D., and Ö.E.Ö. Writing (original draft preparation): A.C.Ö., S.D., and Ö.E.Ö. Writing (review and editing): A.C.Ö., V.U., and Ö.E.Ö. Supervision: A.C.Ö. Project administration: A.C.Ö.

## Funding

No funding was received for this manuscript.

## Data Availability

Further information is available by contacting the corresponding author Ali Cenk Özay (dr.alicenk@gmail.com).
